# The Evidence-Informed Policy Network (EVIPNet) in Chile: lessons learned from a year of coordinated efforts

**DOI:** 10.26633/RPSP.2017.36

**Published:** 2017-03-23

**Authors:** Cristián Mansilla, Cristian A Herrera, Andrea Basagoitia, Tomás Pantoja

**Affiliations:** 1 Ministry of Health of Chile Ministry of Health of Chile Ministry of Health of Chile; 2 Department of Family Medicine Pontificia Universidad Católica de Chile Santiago Chile Department of Family Medicine. Pontificia Universidad Católica de Chile, Santiago, Chile.

**Keywords:** Health policy, planning and management, public health policy, policy making, strategies, health systems, Chile, Health policy, planning and management, public health policy, policy making, strategies, health systems, Chile, Políticas, planejamento e administração em saúde, política de saúde, formulação de políticas, Chile

## Abstract

Informing the health policymaking process with the best available scientific evidence has become relevant to health systems globally. Knowledge Translation Platforms (KTP), such as the World Health Organization’s Evidence Informed Policy Networks (EVIPNet), are a recognized strategy for linking research to action.

This report describes the experience of implementing EVIPNet in Chile, from its objectives, organizational structure, strategy, activities, and main outputs, to its evolution over the course of its first year. Lessons learned are also covered.

Of the activities initiated by EVIPNet-Chile, the Rapid Response Service proved to be a good starting point for engaging policymakers. Capacity building workshops and policy dialogues with relevant stakeholders were also successful. Additionally, EVIPNet-Chile developed a model for engaging academic institutions in policymaking through a network focused on preparing evidence briefs. A number of challenges, such as changing methods for producing rapid evidence syntheses, were also identified. This KTP implementation model located in a Ministry of Health could contribute to the development of similar initiatives in other health systems.

## RESUMEN

Para los sistemas de salud a nivel mundial se ha vuelto cada vez más importante contar con la mejor evidencia disponible como información para el proceso de formulación de políticas de salud. Las plataformas de traducción del conocimiento, como la Red de Políticas Informadas por la Evidencia (EVIPNet, por su sigla en inglés) de la Organización Mundial de la Salud, son estrategias reconocidas para vincular la investigación a la acción.

En este informe se describe la experiencia de la utilización de EVIPNet en Chile, sus objetivos, estructura orgánica, estrategia, actividades y resultados principales de su evolución en el curso de su primer año. Se incluyen asimismo las enseñanzas extraídas.

De las actividades iniciadas por EVIPNet en Chile, el servicio de respuesta rápida resultó ser un buen punto de partida para interesar a los responsables de las políticas. También fueron exitosos los talleres que se llevaron a cabo sobre creación de capacidades y los diálogos de política con los interesados directos pertinentes. Además, EVIPNet en Chile elaboró un modelo para invitar a instituciones académicas a participar en el proceso de formulación de políticas por medio de una red centrada en la preparación de resúmenes de datos científicos. Se encontraron también varios retos, como el cambio de métodos para producir síntesis rápidas de datos científicos. Este modelo de aplicación de plataformas de traducción del conocimiento, ubicado en un Ministerio de Salud, podría contribuir al desarrollo de iniciativas similares en otros sistemas de salud.

## RESUMO

Fundamentar o processo de formulação de políticas de saúde com as melhores evidências científicas disponíveis tornou-se indispensável nos sistemas de saúde em todo o mundo. As plataformas de tradução de conhecimento, como as Redes de Políticas Informadas por Evidências (EVIPNet) da Organização Mundial da Saúde (OMS), são parte de uma estratégia comprovada para vincular a pesquisa à ação.

Este informe descreve a experiência de implantação da EVIPNet no Chile: dos objetivos, estrutura organizacional, estratégia, atividades e principais resultados à evolução ao longo do primeiro ano de atividade. As lições aprendidas são também apresentadas.

Das atividades iniciadas pela EVIPNet-Chile, o Serviço de Resposta Rápida mostrou ser um bom ponto de partida para atrair a participação dos formuladores de políticas. Os seminários de capacitação e os colóquios sobre políticas com os interessados relevantes renderam bons resultados. Além disso, a EVIPNet-Chile elaborou um modelo para atrair a participação das instituições acadêmicas na formulação de políticas com uma rede dedicada ao preparo de resumos de evidências. Um dos muitos desafios identificados é modificar os métodos para produzir sínteses rápidas de evidências. Este modelo de implantação da plataforma de tradução de conhecimento sediado em um ministério da saúde poderia contribuir para a elaboração de iniciativas semelhantes em outros sistemas de saúde.

Decision makers use a number of different inputs to address a wide range of questions about the relevance and size of a policy issue, the impacts of different policy options, and the implementation of these options in their health systems. Research evidence is one of these key inputs in the policymaking process, and could contribute to making better decisions. Although efficient use of available resources has special relevance for Lowand Middle-Income Countries (LMICs), it also pertains to High-Income Countries (HICs) where policymakers strive to make the best use of resources.

In this context, the World Health Organization (WHO) 2004 Annual Report included a chapter on the need for linking research to action (1). Later, in May 2005, the World Health Assembly encouraged countries to “establish or strengthen mechanisms of knowledge-transfer to support public health development, health-related policies, and evidence-based health systems” (2). Afterwards, this call was reinforced by the 2008 Bamako “Call to Action on Research for Health” (3), and more recently, by the 2013 World Health Report (4). The 2013 report issued a strong call for “closer collaboration between researchers and policymakers, i.e., research needs to be taken outside the academic institutions and into public health programs that are close to the supply of and demand for health services” (4)—in order to obtain Universal Health Coverage.

Despite these global calls to use scientific knowledge in policymaking, research evidence has not been systematically used to make recommendations (5). Although policymakers are actually using the available evidence to make decisions, there is still a gap between the availability of scientific knowledge and its systematic use across different levels of the health system, including the policymaking process (6).

Evidence Informed Health Policymaking (EIHPM) aims to ensure that the decision-making process is systematically and transparently informed by the best available scientific evidence (7). The community and civil society could also become part of EIHPM by representing stakeholders in a variety of health policies, transforming policymaking into a participative process (8).

## KNOWLEDGE TRANSLATION PLATFORMS AND EVIPNET

Knowledge Translation (KT), defined as “a dynamic and iterative process including synthesis, dissemination, exchange, and application of knowledge in order to improve population health, provide more effective health services and products, and strengthen health systems,” is closely related to EIHPM (9). In this sense, the development and implementation of Knowledge Translation Platforms (KTP)—partnerships among policymakers, researchers, civil society organizations, and other stakeholders that promote the use of evidence in policymaking—provide the infrastructure for country-level efforts to link research to action (10).

A number of experiences in LMIC countries—e.g., Evidence Informed Policy Network (EVIPNet) in Cameroon (11), Regional East African Community Health Policy Initiative (REACH-PI) in Uganda (11), and Zambia Forum for Health Research (ZAMFOHR) in Zambia (12)—and in high-income countries—e.g., McMaster Health Forum in Canada (13), Center on Knowledge Translation for Disability and Rehabilitation Research (KTDRR) in the United States of America (14), and EVIPNet in Europe (15)—have shown the feasibility of implementing this type of initiative. The EVIPNets are KTPs sponsored by WHO with variable levels of activity across the different WHO Regions. EVIPNet promotes partnerships at the country level among policymakers, researchers, and civil society, in order to facilitate both policy development and implementation using the best scientific evidence available. EVIPNet comprises networks that bring together country-level teams that are coordinated at both the regional and global levels.

## PROMOTING USE OF EVIDENCE FOR POLICYMAKING IN CHILE

Chile has some experience using evidence in policymaking. The processes of formulating and evaluating the National Health Objectives for 2000–2010 (16) and the National Health Plan for 2011–2020 (17) were informed by local and international evidence. In 2004, a law that established “Explicit Guarantees in Health” for 80 prioritized health conditions took into account local prevalence and incorporated specific guidelines for clinical practice for each condition. In 1997, the Ministry of Health (MoH) created a Health Technology Assessment (HTA) unit. In 2012, a National Committee was officially formalized to propose an institutionalization plan for HTA. Currently, this Committee is cooperating with the HTA unit to expand its scope to develop evidence-based recommendations for coverage decisions through the recently created Financial Protection System for High Cost Diagnostics and Treatments.

Between 2010 and 2013, the Secretariat of EVIPNet Americas organized a number of training activities in this WHO Region, aiming to promote EIHPM and to build capacity on preparing evidence briefs for policy (18) and organizing policy dialogues (19). Some of these activities were carried out and coordinated by a group of Chilean researchers interested in the field (20).

Despite these initiatives, the scientific evidence was still not being used systematically in every policy decision in Chile. Moreover, research was not always an important input for decision makers and health policy. Finally, in 2014, the MoH opened a full-time position exclusively dedicated to establishing an EVIPNet working group in Chile.

This paper describes the development and evolution of EVIPNet-Chile and identifies some of the lessons learned and challenges met after a year of experience. It describes the process of developing this initiative within the MoH, including objectives, organizational structure, strategy, activities, and main outputs during the period from October 2014 to October 2015.

## EVIPNET-CHILE

### Governance, structure, and stakeholders

The structure of a KTP within a country could have an important impact for EIHPM. However, there is not a unique “organizational solution” to where the platform should be located in the national health system. There are examples of KTPs in universities, non-governmental organizations, government institutions, and public agencies (21).

**FIGURE 1.**
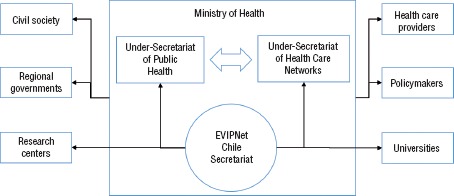
Governance, structure, and stakeholders of the Evidence-Informed Policy Network (EVIPNet)-Chile, 2015

***Source:*** Prepared by the authors from the study data.

EVIPNet-Chile is coordinated by a Secretariat hosted by the Cabinet of the Minister at the MoH. This hosting enables a direct connection with the highest authorities of the MoH and other secretariats, with policymakers, local governments, public health care providers, civil society, and other stakeholders in health policymaking. In addition, EVIPNet-Chile Secretariat facilitates a fluid dialogue with universities and research groups (Figure 1).

As a first step, in 2015, EVIPNet-Chile initiated a partnership with the Network of Schools and Departments of Public Health at a number of universities in Chile. This partnership—called the EVIPNet-Chile Network—is supported by the Country Office of the Pan American Health Organization (PAHO). It aims to expand the capacity of EVIPNet-Chile, to organize activities and prepare evidence syntheses to policymakers, and increase the numbers of those trained (critical mass) in EIHPM concepts across the country.

### Strategy

EVIPNet-Chile aims to support the health policymaking process, in order to improve public health and reduce health inequities in Chile. It has three main strategic objectives:

- To use the best available evidence to produce outputs that can inform decisions in the health policymaking process.
- To promote the systematic use of evidence in the health policymaking process.
- To promote collaboration among policymakers, researchers, and civil society organizations.

In order to accomplish these objectives, EVIPNet-Chile has outlined five main types of activities/products (Table 1):

- Rapid Response Service for rapid evidence synthesis
- Website (one-stop shop)
- Capacity-building workshops
- Evidence briefs for policy
- Policy dialogues

The overall strategy of EVIPNet-Chile is organized in a matrix where each activity/product is aligned with each objective. The matrix shown in Table 2 is a visual display of the relationship between objective and activity/product. This matrix allows identification of a portfolio of activities/products to address specific objectives. For instance, when a team is particularly interested in promoting collaboration among policy-makers, researchers, and civil society, its time would be best invested in writing rapid evidence syntheses, preparing evidence briefs for policy, and/or organizing policy dialogues, rather than website development or capacity-building work-shops. This matrix also allows more efficient monitoring and evaluation of EVIPNet-Chile activities, allocating indicators, results, and targets to each activity–objective.

**TABLE 1.** Description of Evidence-Informed Policy Network (EVIPNet)-Chile activities/products developed to better inform health policy decision-making, Chile, 2015

	Activities	Description
i	Rapid Response Service	A service that systematically responds to urgent evidence needs within the Ministry of Health. It aims to balance opportunity (urgency) with the depth of the synthesis.
ii	Website (one-stop shop):	Quick access to several tools and evidence for better informed health decisions.
iii	Capacity building workshops	Training sessions for Ministry of Health professionals aimed to improve current capacities in evidence-informed health policymaking.
iv	Evidence briefs for policy	A relatively new form of research synthesis where the best available global research evidence, such as systematic reviews, and relevant local data and studies are synthesized to clarify the problems associated with the issue, describe what is known about options resolving these, and identify key considerations for implementing each option.
v	Policy dialogues	Activities that facilitate interaction among researchers, policymakers, and stakeholders. These consider the best available global and local research evidence, along with the tacit knowledge of the key health system “actors,” who are either involved in the issue or likely to be affected by the decision/outcome.

***Source:*** Prepared by the authors from the study data.

**TABLE 2.** Evidence-Informed Policy Network (EVIPNet)-Chile matrix showing the relationship between strategic objectives and activities, where a checkmark indicates the activity/product addresses the objective directly, Chile, 2015

Strategic objectives			Activities/products	
	Rapid response service	Website	Capacity.building workshops	Evidence briefs for policy	Policy dialogues
a)	To use the best available evidence to produce outputs that can inform decisions in the health policymaking process	✓			✓	
b)	To promote the systematic use of evidence in the health policymaking process		✓	✓		✓
c)	To promote collaboration among policymakers, researchers, and civil society organizations	✓			✓	✓

***Source:*** Prepared by the authors from the study data

### Activities

Although there is a variety of existing activities in which a KTP might engage, in Chile the aforementioned five were chosen as a good starting point, and a balanced combination that would address all the strategic objectives.

**FIGURE 2.**
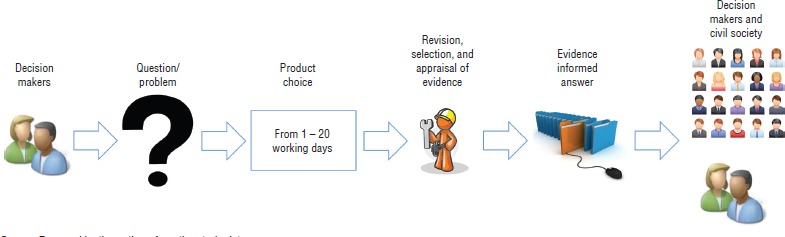
Process to create a rapid evidence synthesis, a main activity of the Evidence-Informed Policy Network (EVIPNet)-Chile, 2015

*Rapid Response Service (preparing rapid evidence syntheses).* On a daily basis, decision makers require urgent evidence-informed answers to a number of policy questions. The Rapid Response Service aims to improve evidence accessibility to them by significantly reducing the time needed to summarize the existing research. The EVIPNet-Chile Secretariat prepares rapid evidence syntheses (summaries of the impact of specific interventions) in less than 20 working days, based mainly on relevant systematic reviews. The depth of the evidence analysis depends on the time available to deliver the product (Figure 2).

Although the types of questions that the Rapid Response Service answers are mainly related to the impact of health policies or health system interventions, decision makers’ needs are frequently broader. Therefore, many requirements start with an unstructured question that needs to be clarified and framed before the Rapid Response Service can provide an answer. In its first 12 months of operation, the Rapid Response Service received and responded to 23 questions from various departments within the MoH, addressing a number of different policy issues (Table 3).

The process of preparing a rapid evidence synthesis shares several steps with that of preparing a systematic review (22). However, there is not an agreed-upon method to write a rapid evidence synthesis, and methodological approaches vary considerably (23–25). The methods used by the EVIPNet-Chile Secretariat to produce rapid evidence syntheses have been continuously modified to consider the specific challenges faced in preparing each of them. Nonetheless, the process of developing a handbook is underway and will provide a step-by-step guide to produce this type of product systematically.

*Website (one-stop shop).* One-stop shops are a useful strategy for improving accessibility to research evidence by health policymakers. There are a number of successful initiatives in this area, such as Health Systems Evidence in Canada (26), PDQ-Evidence (27), and CIPHER in Australia (28).

**TABLE 3.** List of rapid evidence syntheses prepared by the Evidence-Informed Policy Network (EVIPNet)-Chile, 2015 Synthesis topic

Synthesis topic	Description	Working days	Requested by
Defibrillators	Impact of a public access defibrillation program	5	Cabinet of the Minister
Human milk banks	Impact of human milk banks	10	Cabinet of the Minister
Food labeling	Joint impact of three policies: food-labelling, food advertising restriction, and food sell restriction	15	Public Policies Division
Primary care receptionist	Impact of a receptionist in a primary care center on patient satisfaction	5	Cabinet of the Minister
Female condom	Impact of a policy to promote female condom	15	Prevention and Disease Control Division
Sugar food tax	Impact of a sugar-sweetened solid food tax	7	Public Policies Division
Condom dispensers	Impact of condom dispensers in public places	5	Cabinet of the Minister
Omega-3 fatty acids	Benefits of Omega-3 fatty acids	15	Public Policies Division
Acupuncture	Describe implementation strategies for acupuncture in a primary care setting	10	Public Policies Division
Menthol in cigarettes	Impact of a restriction of menthol in tobacco cigarettes	10	Public Policies Division
Financial mechanisms in primary care	Describe possible indicators, in order to adjust the financial mechanism for communal primary care	5	Primary Care Division
Medical leave for parents	Impact of a medical leave for parents with severe ill children	5	Prevention and Disease Control Division
Probiotics	Safety of probiotics in infants formula	10	Public Policies Division
Medical use of cannabis	Benefits of medicinal use of cannabis	10	Cabinet of Undersecretary of Public Health
Medical Loss Ratio	Impact of setting a Medical Loss Ratio in private health insurance market	5	Cabinet of the Minister
MRP vaccine	Risk of Measles-Rubella-Parotitis vaccine in adults	5	Prevention and Disease Control Division
Pharmaceutical market	Impact of market competition in pharmaceutical industry	5	Cabinet of the Minister
Daylight Saving Time	Impact of the daylight saving time setting	4	Cabinet of Undersecretary of Public Health
Sexual health & indigenous people	Educational interventions in sexual and reproductive health for indigenous people	10	Prevention and Disease Control Division
Births and hospitals	Risk of planned births in settings other than hospitals	10	Prevention and Disease Control Division
Water supply	Water supply mechanism for isolated coastal communities	5	Public Policies Division
Cannabis smoking	Benefits and risks of smoking marijuana	15	Cabinet of Undersecretary of Public Health
Dental dams	Risk of using dental dams to prevent sexually transmitted infections	5	Prevention and Disease Control Division

***Source:*** Prepared by the authors from the study data.

Following EVIPNet Global templates, EVIPNet-Chile developed a website accessible from within the MoH. This website provides access to the rapid evidence syntheses prepared by the team (Table 3), as well as evidence briefs for policy, policy dialogue summaries, and workshop presentations. In addition, the EVIPNet-Chile website allows users to access relevant KT resources, such as news, publications, multimedia, and events, and offers a specialized search engine for locating relevant literature. There is a slightly different version of the website accessible from outside the MoH. From its launch in February 2015, through October 2015, the EVIPNet-Chile website had been visited more than 1000 times from within Chile alone.

***Capacity-building workshops.*** Since 2014, EVIPNet-Chile has organized four half-day workshops within the MoH, and one with the EVIPNet-Chile Network. These workshops are designed to build capacity on the systematic and transparent use of evidence in policymaking. There are two types of workshops:

- Driven by the EVIPNet-Chile Secretariat: workshops that address the general concepts of EIHPM and the use of systematic reviews. These workshops are built mainly from contents developed by the team at the McMaster Health Forum (13).
- User-demanded: workshops that address specific training needs. For example, methods to produce rapid evidence syntheses, requested by the HTA Unit at the MoH, and methods for preparing evidence briefs for policymakers in the EVIPNet-Chile Network.

These workshops have reached more than 30 of the almost 50 eligible professionals at the MoH and 20 members of EVIPNet-Chile Network across the country.

***Preparation of evidence briefs for policy.*** These evidence summaries take a policy problem/issue as a starting point, describe its underlying factors, and frame a number of relevant policy options while identifying barriers and facilitators to their implementation. Then the focus turns to finding and distilling the full range of research evidence relevant to the various features of the problem/issue, such as the impact of the different options or the effectiveness of implementation strategies (18).

The problem identified for an evidence brief should be broad and also, a political priority. In addition, it should be developed together with the MoH “issue-owner,” i.e., those charged with managing the issue.

As mentioned before, participation in the EVIPNet-Chile Network is a key element in this process. Evidence briefs for policy are mainly prepared by academic teams that have attended a capacity-building workshop organized by the EVIPNet-Chile Secretariat. The PAHO Country Office has also played a crucial role in this partnership by supporting the preparation of these products. The policy issues addressed by the evidence briefs for policy are related to the MoH priorities and communicated to the network by the EVIPNet-Chile Secretariat.

***Organizing policy dialogues.*** Structured discussions about an evidence brief for policy or a rapid evidence synthesis can contribute to the development of evidence-informed health policies, helping to clarify the problem and solutions, and to develop a shared understanding among stakeholders (19, 29).

Over the course of the first year, EVIPNet-Chile organized three policy dialogues based on three rapid evidence syntheses: human milk banks, female condoms, and acupuncture (see Table 3). Health care providers, ministries, non-governmental organizations (associations of patients and workers), academic institutions, and scientific societies have attended these meetings, contributing to the deliberations and adding inputs to the process, beyond scientific evidence.

## DISCUSSION

Implementing KTPs is an effective strategy to inform decision-making processes with evidence, providing an efficient route to improving health policies, especially in LMICs (30). EVIPNet-Chile provides a clear experience of how a KTP can be established and institutionalized within a MoH and is a good example of a working KTP with a portfolio of different activities and products. After this first year, lessons learned from the experience in Chile can be summarized in five main points.

First, EVIPNet-Chile provides a concrete example of how a KTP can be established and institutionalized within a MoH. This model has allowed a closer relationship with policymakers and more permanent relationships within the Government, a main objective of a KTP (21).

Second, the balanced portfolio of initiatives that a KTP decides to incorporate is very important to comprehensively addressing its objectives. Although EVIPNet-Chile has defined five main activities/products, there are others that could be used to further develop EIHPM, such as summaries of systematic reviews (31), citizen panels (32), and communities of Practice (33).

Third, the Rapid Response Service has been the most used and valued activity in our MoH because it is perceived as providing timely evidence for policy-making. A rapid evidence synthesis is a very efficient instrument for engaging policymakers with KT, since it quickly gets an evidence-informed answer to a specific policy question. In this sense, Rapid Response Services can be a very good starting point when introducing a KTP within a MoH. Although EVIPNet-Chile has used mainstream methods, mainly based on the use of systematic reviews, the specific processes have continuously changed over the study period. We are now using rapid evidence syntheses as an input for policy dialogues; this represents an innovation to prior KT research (19, 29).

Fourth, collaboration with academic groups arose from the EVIPNet-Chile Secretariat’s need for human resources; previously, their availability was not enough to prepare evidence briefs for policy. A lack of resources for any KTP, especially initially, can be used as an opportunity to expand the network by engaging other stakeholders in the process.

Lastly, it is important for KTP professionals to be up-to-date in terms of current technologies and methodologies that could be used to better link research to action in policymaking. The EVIPNet-Chile Secretariat has been paying close attention to new developments and innovations in this area. Some examples are Epistemonikos (evidence matrix systematic reviews with primary studies cited) (27), Health Systems Evidence new web interface (34) (enhanced features), and RevMan (to develop meta-analysis for rapid evidence synthesis) (35).

### Barriers and challenges

There are important barriers that we addressed during this first year. For instance, although the EVIPNet-Chile Network has been positively evaluated to date, it is necessary to consolidate the relationships among KT, the policymaking process, and academic institutions. This network has been a major advance in terms of preparing evidence briefs for policy, but the work could be expanded into other new activities/products in the future.

Secondly, as a new program inside a Ministry, it is necessary to make a cultural change for promoting the use of evidence in policymaking processes. This is generally a hard process, considering the existing different uses of evidence. In this sense, Rapid Response Service has been a useful tool, consolidating an important position within the MoH.

Lastly, uses of evidence in the different stages of the policymaking process have also been challenging. The balanced combination of rapid evidence syntheses, evidence briefs for policy, and policy dialogues has successfully responded to the needs of policymakers and relevant stakeholders in a variety of scenarios and stages of the policymaking process.

A number of challenges should be considered when implementing a KTP. First, as a part of the KT process, it is necessary to have a plan for incorporating new developments on EIHPM. For example, text mining (36) and living evidence (37) are current evolving topics worth incorporating in the foreseeable future in our portfolio of methods. Acting on what has been learned from past experiences with evidence briefs is also imperative (38).

### Conclusions and recommendations

A KTP can certainly be enriched by collaboration with international organizations, such as PAHO and WHO, but also by engaging with other countries of Latin America that are working on similar initiatives. Also, closer contact with non-governmental organizations working in this field, such as the Cochrane Collaboration, would be a major improvement, especially in terms of developing methodologies. The Rapid Response Service was the most challenging part of this effort because there is no standard method for producing the summaries; regardless, the service has been widely demanded within the MoH.

Finally, in order to concretely evaluate the impact of EVIPNet-Chile on the policymaking process, it is necessary to conduct rigorous evaluations. A good starting point could be an analysis of the strategy matrix presented in Table 2, defining indicators related to each activity. Such analysis would determine how well the objectives are being addressed by the work of EVIPNet-Chile, and how its implementation has specifically improved evidence-informed policymaking in our health system.

### Disclaimer.

Authors hold sole responsibility for the views expressed in the manuscript, which may not necessarily reflect the opinion or policy of the *RPSP/PAJPH* and/or PAHO.
